# Whole-genome sequence of *Streptococcus agalactiae* strain GIFTS31 isolated from streptococcosis-infected Nile tilapia in Bangladesh

**DOI:** 10.1128/mra.00441-26

**Published:** 2026-08-18

**Authors:** Santu Biswas, Md. Rony Babu, Promi Saha, Farzana Yeasmin, Arpita Guha, Md. Amzad Hossain, Md. Mustafizur Rahman, Tofazzal Islam, Md. Mahbubur Rahman

**Affiliations:** 1 Institute of Biotechnology and Genetic Engineering, Gazipur Agricultural University 198780, Gazipur, Bangladesh; 2 Department of Aquaculture, Gazipur Agricultural University 198780, Gazipur, Bangladesh; 3 Infectious Diseases Division, International Centre for Diarrhoeal Disease Research Bangladesh 56291, Dhaka, Bangladesh; University of Maryland School of Medicine, Baltimore, Maryland, USA

**Keywords:** *Streptococcus agalactiae*, tilapia, streptococcosis, virulence genes, antimicrobial resistance gene

## Abstract

*Streptococcus agalactiae* strain GIFTS31 was isolated from a Nile tilapia infected with streptococcosis in Gazipur, Bangladesh. The draft genome of GIFTS31 comprises 2,039,674 bp with a GC content of 35% and encodes 1,957 predicted protein-coding sequences. The genome sequence provides valuable insights into the pathogenic potential of fish-associated *S. agalactiae*.

## ANNOUNCEMENT

Streptococcosis, caused by *Streptococcus agalactiae* and other species, is a major bacterial disease of warm-water aquaculture (1–3), affecting tilapia and several other fish species and causing substantial economic losses worldwide (1–5). Here, we report the draft genome sequence of fish-pathogenic *S. agalactiae* strain GIFTS31.

GIFTS31 was isolated from the eye of a Nile tilapia exhibiting typical clinical signs of streptococcosis in a fish farm in Gazipur district of Bangladesh (24.110312° N, 90.325237° E). Infected fish showed bilateral exophthalmia, corneal opacity, diffuse hemorrhages in the skin and fin bases, anorexia, and spiraling swimming behavior. Bacterial isolation was performed using *S. agalactiae* selective agar base supplemented with 6% sheep blood and incubated at 28°C for 48 h. The pure (cream-whitish, circular shaped) colony of GIFTS31 was obtained after repeated streaking on the same media and identified as *S. agalactiae* based on its colony morphology and characteristic biochemical properties (1).

Genomic DNA was extracted from an overnight culture grown in Todd Hewitt broth at 37°C using the DNeasy Blood & Tissue Kit (QIAGEN). DNA quality and quantity were assessed using a NanoDrop (Thermo Fisher Scientific, USA) and a Qubit Fluorometer (Thermo Fisher Scientific), and DNA fragment quality was evaluated using a LabChip GX Bioanalyzer (PerkinElmer). Sequencing libraries were prepared using the Illumina DNA Prep Kit and sequenced on an Illumina NextSeq 2000 platform using a P1 flow cell (2 × 150 bp paired-end reads).

A total of 1,287,920 paired-end raw reads were obtained. Read quality was assessed using FastQC v0.12.1 (6), followed by trimming with Trimmomatic v0.39 (7). High-quality reads were assembled *de novo* using SPAdes v4.2.0 (8) with Phred+33 quality encoding. Genome annotation was performed using the NCBI Prokaryotic Genome Annotation Pipeline v6.10 (9). Genome completeness was assessed using CheckM v1.2.4 (10). Taxonomic classification was confirmed by NCBI average nucleotide identity (ANI) analysis (11). Antibiotic-resistance genes (ARGs) were identified using CARD (12) and ResFinder v4.7.2 (13), while virulence-associated genes (VFGs) were predicted using VFDB (14). Plasmid replicons were screened using PlasmidFinder-2.0 (15). Functional subsystem analysis was performed using the RAST v2.0 server (16). Default parameters were used for all software unless otherwise speciﬁed.

The genomic features of *S. agalactiae* strain GIFTS31 are summarized in Table 1. The draft genome consists of approximately 2 Mbp with a GC content of 35%, exhibiting 98.08% completeness and 0.13% contamination. ANI analysis demonstrated 98.99% identity with the genome of *Streptococcus agalactiae* type strain (GCA_900458965.1), confirming its taxonomic classification. The genome contains 1,957 predicted protein-coding sequences and 233 functional subsystems associated with metabolism, stress response, and virulence. CARD and ResFinder identified four ARGs (*vanT*, *VanY*, *mprF*, and *mreA*) associated with antibiotic resistance. VFDB identified 52 virulence-associated genes responsible for adherence (*lmb*, *fbsB*, *fbp5A*, pilus island genes, and *gapA*), enzymes (*hylb* and *eno*), immune evasion (cps cluster), immunoreactive antigens (*rib* and *sip*), and toxins (cytolysin genes, *cfa*/*cfb*), highlighting the pathogenic potential of the strain. Two CRISPR arrays were detected, whereas no plasmid replicon sequences were identified.

**TABLE 1:** Genomic features of *S. agalactiae* strain GIFTS31, isolated from disease-infected tilapia fish

Genomic features	*Streptococcus agalactiae* GIFTS31
GenBank accession no.	JBSXVX000000000
BioSample accession no.	SAMN53801996
SRA accession no.	SRR36540872
Assembled genome size (bp)	2,039,674
No. of reads generated (raw reads)	1,287,920
GC%	35
Coverage (×)	179.0
Longest contig size	437,038
Shortest contig size	246
Genome completeness (%)	98.08
*N*_50_ value (kbp)	152.7
Number of contigs	36
Genes (total)	2,082
Coding sequences (CDSs) (total)	2,029
Genes (coding)	1,957
CDSs (with protein)	1,957
Genes (RNA)	53
rRNAs	1, 1, 1 (5S, 16S, 23S)
Complete rRNAs	1, 1, 1 (5S, 16S, 23S)
tRNAs	46
ncRNAs	4
Pseudogenes (total)	72
CDSs (without protein)	72
Pseudogenes (ambiguous residues)	0 of 72
Pseudogenes (frameshifted)	33 of 72
Pseudogenes (incomplete)	41 of 72
Pseudogenes (internal stop)	19 of 72
Pseudogenes (multiple problems)	16 of 72
CRISPR arrays	2
Number of ARGs	4
Number of VFGs	52
Number of subsystems	233

This genome sequence provides a valuable resource for studying virulence, antimicrobial resistance, and epidemiology of fish-associated *S. agalactiae*.

## Data Availability

The whole-genome shotgun projects of the *Streptococcus agalactiae* strain GIFTS31 have been deposited in GenBank under accession number JBSXVX000000000. The versions described in this paper are the first versions. The raw sequencing reads have been deposited in the NCBI Sequence Read Archive under accession number SRR36540872, associated with BioSample accession number SAMN53801996.

## References

[1] Abdallah ESH, Metwally WGM, Abdel-Rahman MAM, Albano M, Mahmoud MM. 2024. *Streptococcus agalactiae* infection in Nile tilapia (*Oreochromis niloticus*): a review. Biology (Basel) 13:914. doi:10.3390/biology13110914 39596869 PMC11591708

[2] Foysal MJ, Alam M, Momtaz F, Chaklader MR, Siddik MAB, Cole A, Fotedar R, Rahman MM. 2019. Dietary supplementation of garlic (*Allium sativum*) modulates gut microbiota and health status of tilapia (*Oreochromis niloticus*) against *Streptococcus iniae* infection. Aquac Res 50:2107–2116. doi:10.1111/are.14088

[3] Choresca CH Jr, Legario FS, De Leon MEE, Santos MNM, Bumanlag BE, Ca-As CGP, Gente AA, Gloria PCT. 2025. Draft genome of *Streptococcus agalactiae* serotype Ia FBC260 causing hemorrhagic septicemia with massive cellular meningitis in cultured Nile tilapia from the Philippines. Microbiol Resour Announc 14:e00911-24. doi:10.1128/mra.00911-24 39651875 PMC11737160

[4] Amal MNA, Zamri-Saad M, Iftikhar AR, Siti-Zahrah A, Aziel S, Fahmi S. 2012. An outbreak of *Streptococcus agalactiae* infection in cage-cultured golden pompano, *Trachinotus blochii* (Lacépède), in Malaysia. J Fish Dis 35:849–852. doi:10.1111/j.1365-2761.2012.01443.x 22913387

[5] Chen B, Xu M, Li F, He Z, Ouyang P, Chen D, Geng Y, Huang X. 2025. Pathological and molecular characteristics of a new infectious *Streptococcus agalactiae* from farmed Schizopygopsis pylzovi in southern China. Aquaculture 594:741407. doi:10.1016/j.aquaculture.2024.741407

[6] Andrews S. 2010. FastQC: a quality control tool for high throughput sequence data. Babraham Bioinformatics, Babraham Institute, Cambridge, United Kingdom. https://www.bioinformatics.babraham.ac.uk/projects/fastqc.

[7] Bolger AM, Lohse M, Usadel B. 2014. Trimmomatic: a flexible trimmer for Illumina sequence data. Bioinformatics 30:2114–2120. doi:10.1093/bioinformatics/btu170 24695404 PMC4103590

[8] Prjibelski A, Antipov D, Meleshko D, Lapidus A, Korobeynikov A. 2020. Using SPAdes *de novo* assembler. Curr Protoc Bioinformatics 70:e102. doi:10.1002/cpbi.102 32559359

[9] Tatusova T, DiCuccio M, Badretdin A, Chetvernin V, Nawrocki EP, Zaslavsky L, Lomsadze A, Pruitt KD, Borodovsky M, Ostell J. 2016. NCBI prokaryotic genome annotation pipeline. Nucleic Acids Res 44:6614–6624. doi:10.1093/nar/gkw569 27342282 PMC5001611

[10] Parks DH, Imelfort M, Skennerton CT, Hugenholtz P, Tyson GW. 2015. CheckM: assessing the quality of microbial genomes recovered from isolates, single cells, and metagenomes. Genome Res 25:1043–1055. doi:10.1101/gr.186072.114 25977477 PMC4484387

[11] Ciufo S, Kannan S, Sharma S, Badretdin A, Clark K, Turner S, Brover S, Schoch CL, Kimchi A, DiCuccio M. 2018. Using average nucleotide identity to improve taxonomic assignments in prokaryotic genomes at the NCBI. Int J Syst Evol Microbiol 68:2386–2392. doi:10.1099/ijsem.0.002809 29792589 PMC6978984

[12] Alcock BP, Huynh W, Chalil R, Smith KW, Raphenya AR, Wlodarski MA, Edalatmand A, Petkau A, Syed SA, Tsang KK, et al.. 2023. CARD 2023: expanded curation, support for machine learning, and resistome prediction at the Comprehensive Antibiotic Resistance Database. Nucleic Acids Res 51:D690–D699. doi:10.1093/nar/gkac920 36263822 PMC9825576

[13] Bortolaia V, Kaas RS, Ruppe E, Roberts MC, Schwarz S, Cattoir V, Philippon A, Allesoe RL, Rebelo AR, Florensa AF, et al.. 2020. ResFinder 4.0 for predictions of phenotypes from genotypes. J Antimicrob Chemother 75:3491–3500. doi:10.1093/jac/dkaa345 32780112 PMC7662176

[14] Chen L, Zheng D, Liu B, Yang J, Jin Q. 2016. VFDB 2016: hierarchical and refined dataset for big data analysis–10 years on. Nucleic Acids Res 44:D694–D697. doi:10.1093/nar/gkv1239 26578559 PMC4702877

[15] Carattoli A, Zankari E, García-Fernández A, Voldby Larsen M, Lund O, Villa L, Møller Aarestrup F, Hasman H. 2014. *In silico* detection and typing of plasmids using PlasmidFinder and plasmid multilocus sequence typing. Antimicrob Agents Chemother 58:3895–3903. doi:10.1128/AAC.02412-14 24777092 PMC4068535

[16] Overbeek R, Olson R, Pusch GD, Olsen GJ, Davis JJ, Disz T, Edwards RA, Gerdes S, Parrello B, Shukla M, Vonstein V, Wattam AR, Xia F, Stevens R. 2014. The SEED and the Rapid Annotation of microbial genomes using Subsystems Technology (RAST). Nucleic Acids Res 42:D206–D214. doi:10.1093/nar/gkt1226 24293654 PMC3965101

